# IL-23 enhances the malignant properties of hepatoma cells by attenuation of HNF4α

**DOI:** 10.18632/oncotarget.24875

**Published:** 2018-06-19

**Authors:** Qing Jiang, Yuanli Sun, Zilong Guo, Ru Chen, Simin Ma, Mingpeng Fu, Huifen Zhu, Qin Ning, Ping Lei, Guanxin Shen

**Affiliations:** ^1^ Department of Immunology, School of Basic Medicine, Tongji Medical College, Huazhong University of Science and Technology, Wuhan, Hubei, China; ^2^ Department of Allergy, Tongji Hospital, Tongji Medical College, Huazhong University of Science and Technology, Wuhan, Hubei, China; ^3^ Department of Infectious Disease, Institute of Infectious Disease, Tongji Hospital, Tongji Medical College, Huazhong University of Science and Technology, Wuhan, Hubei, China

**Keywords:** IL-23, hepatocyte nuclear factor 4α, HBV, tumor progression

## Abstract

Chronic infection with hepatitis B virus (HBV) is one of the major risk factors for hepatocellular carcinoma. HBV infection can induce the expression of IL-23. However, the effects of IL-23 on carcinogenesis are rare and contradictory. To investigate the potential role of IL-23 on malignant properties of hepatoma cells, in the present study, first, we confirmed that HBV drove infected hepatoma cells to produce more IL-23. And then we found that at low concentration, human recombinant IL-23 (hrIL-23) enhanced malignant properties of hepatoma cells through increasing the proportion of stem/progenitor cells, promoting proliferation and colony formation, reducing apoptosis and inducing motility and invasivity of them. Hepatocyte nuclear factor 4 alpha (HNF4α), which is essential for liver development and hepatocyte function, was found to be downregulated in HBV integrated or transiently transfected hepatoma cells. Its expression was also decreased in cells treated by hrIL-23 or by HepG2.215 culture supernatant and this decrease could be abolished by supplementation of anti-IL-23p19 antibody. Hence, it is speculated that HBV related IL-23 can enhance malignant properties of hepatoma cells through attenuation of HNF4α. The findings identified a potential target of interventional strategies for treating hepatitis B patients through manipulation of the IL-23.

## INTRODUCTION

Hepatocellular carcinoma (HCC) is one of the most common cancers worldwide [1, 2]. Chronic infection with hepatitis B virus (HBV) is one of the major risk factors for HCC and plays an important part in the prevalence of HCC [3–5].

Cytokines can mediate a variety of biological behavior of cells and have a role in the development of HBV-related HCC. It was reported that HBV can upregulate the expression of cytokines and cause liver damage directly through the cytokines signaling pathway. For instance, we previously reported that HBx induced IL-32 expression through NF-κB activation [6]. HBV surface antigen (HBsAg) could efficiently induce IL-23 secretion in a mannose receptor (MR)-dependent manner [7]. HBx induced IL-23 secretion through the activation of the ERK/NF-κB pathway [8]. The baseline level of IL-23 in serum and hepatic tissue was found to be higher in patients with CHB than in controls [8, 9]. IL-23 belongs to the IL-12 cytokine family, which shares the p40 subunit of IL-12 but is distinguished from the latter by its cytokine subunit, p19 [10]. Consistent with the structural and biological similarities of IL-12 and IL-23, IL-23 receptor is composed of the IL-12Rβ1 subunit and the novel IL-23R subunit which is different from IL-12 receptor [10, 11]. IL-23 and its receptor mainly express in monocytes, DCs, T cells, natural killer cells and myeloid cells [11, 12].

IL-23 was reported to play conflictive roles in carcinogenesis. For instance, exogenously overexpressed IL-23 exerts potent antitumor and anti-metastatic effects to inhibit cancer progression [13–16]. However, Langowski *et al.* reported that mice deficient in IL-23p19 were resistant to tumor induction and treated with anti-IL-23p19 show decreased tumor growth and increased tumor rejection [17]. And IL-23 promoted growth and proliferation of human squamous carcinoma cells of the oral cavity [18]. Through its receptor expressed on cancer cells, IL-23 participated in the progress of colorectal cancer [19] and regulated the proliferation of lung cancer in a concentration-dependent manner [20].

Given the important role of HBV in the prevalence of HCC and the upregulation of IL-23 induced by HBV, it merits to investigate if IL-23 could affect the biological behavior of hepatoma cells and, if so, the underlying mechanisms. In this article, we found that IL-23 did enhance the malignant properties of hepatoma cell lines HepG2 and Huh-7. This enhancement promoted hepatoma cells progressing into invasive cell by the attenuation of HNF4α, which is essential for liver development and hepatocyte function [21]. These findings identified potential targets of interventional strategies for treating hepatitis B patients through manipulation of the IL-23.

## RESULTS

### IL-23 expression is elevated in HBV-integrated HepG2.215 cells

**Figure 1:**
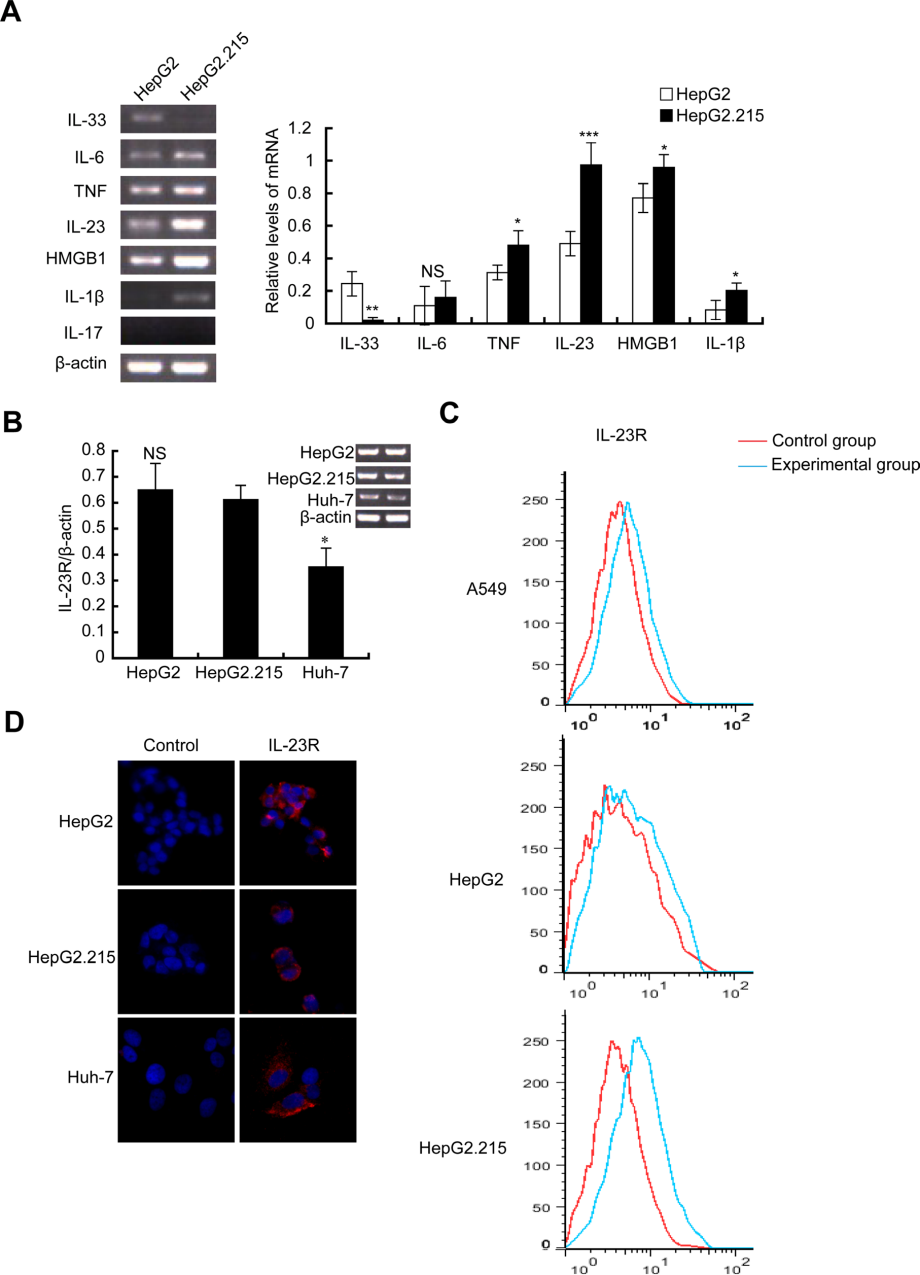
IL-23 expression was elevated in HBV-integrated HepG2.215 cells **(A)** RT-PCR and statistical analysis showed the expression of inflammatory cytokines (IL-1β, IL-6, TNF, IL-23, HMGB1, IL-17 and IL-33) in the hepatoma cell lines HepG2 and HepG2.215. Photograph is one represent of three independent experiments. IL-23R expression was detected by RT-PCR (**B**), flow cytometer (**C**) and immunofluorescence (**D**) in HepG2, Huh-7 and HepG2.215. Hochest33342 was used to stain nuclei. Magnification, ×400. ^*^P < 0.05, ^**^P < 0.01, ^***^P < 0.001, NS, non-significant difference.

Previous studies have revealed the correlation between elevated expression of IL-23 and HBV infection [7–9]. To explore the role of IL-23 in progression of HBV-related HCC, elevated IL-23 expression was confirmed in hepatoma cell lines HepG2 and HBV-integrated HepG2.215 cells. As shown in Figure 1A, the mRNA levels of inflammatory cytokines (such as TNF, IL-23, HMGB1, IL-1β) in HepG2.215 cells were higher than those in HepG2 cells. Among them, IL-23 increased more evidently. We then evaluated the expression of IL-23 receptor (IL-23R) on these cells lines. RT-PCR results showed that the mRNA of IL-23R could be detected in HepG2, HepG2.215 and Huh-7 cells. The mRNA level of IL-23R showed no statistically difference between HepG2 and HepG2.215 (Figure 1B) but was decreased in Huh-7 (*P* < 0.05). Flow cytometry results (Figure 1C) manifested that IL-23R expression levels on HepG2 and HepG2.215 cells were parallel to that on A549 cells which were reported to show strong positive expression of the IL-23R [20]. Immunofluorescence staining confirmed positive expression of IL-23R on these 3 hepatoma cell lines (Figure 1D). Expression of the IL-23R in liver cancer cells inferred that hepatoma cells might be the potential targets of IL-23.

### hrIL-23 enhances growth of hepatoma cells

**Figure 2:**
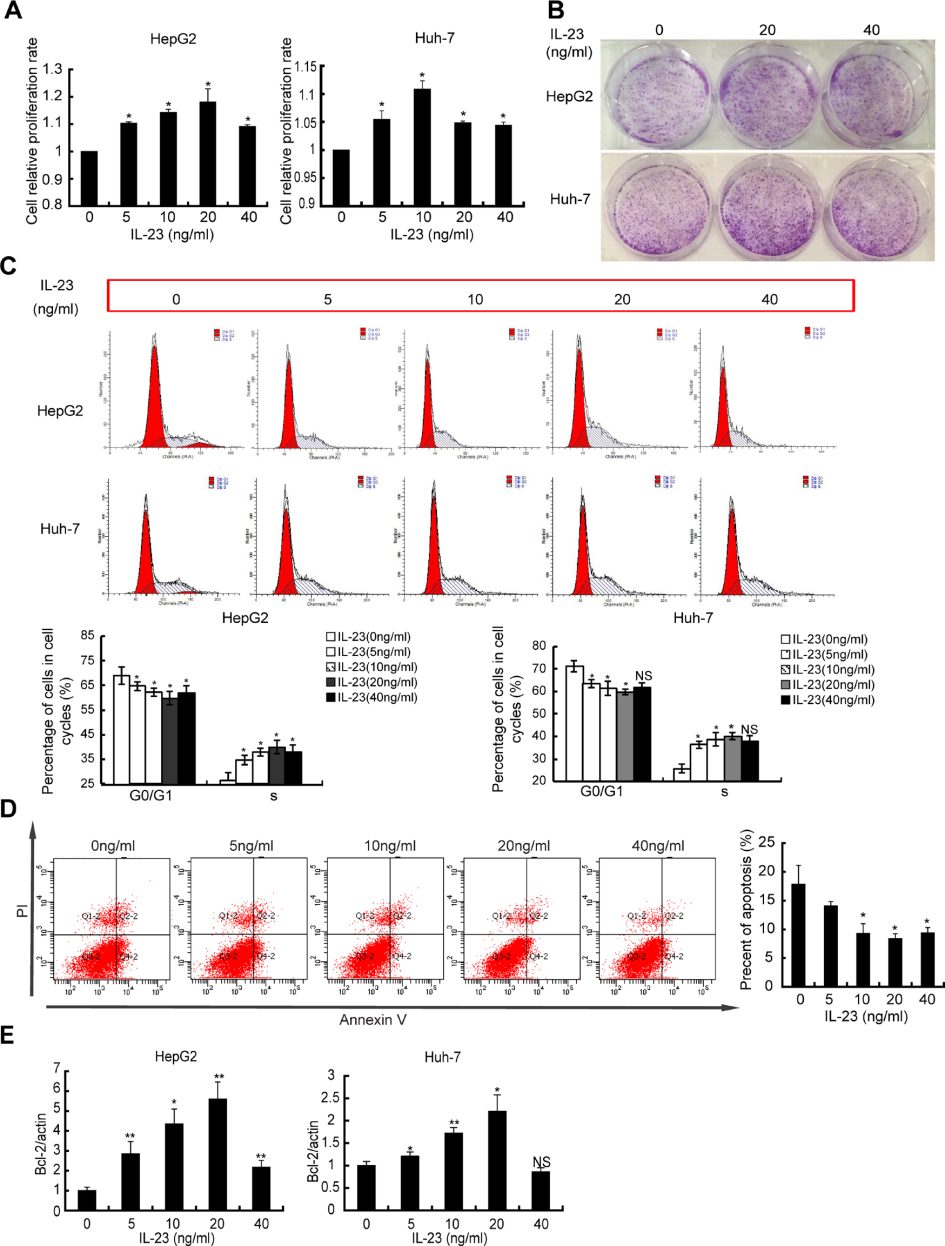
hrIL-23 regulates growth of hepatoma cells Cells were treated with hrIL-23 (0 ng/ml, 5 ng/ml, 10 ng/ml, 20 ng/ml, 40 ng/ml) for 48 h. (**A**) CCK8 proliferation assay was performed. Data were expressed as fold change in proliferation rate in hrIL-23-treated cells versus untreated ones (arbitrary value = 1). Each value represents the mean ± SD for triplicate samples. (**B**) Cells were seeded at 500 cells/cm^2^ and incubated for 5–7 days to form colonies. A representative experiment was shown. The experiment was performed two times yielding similar results. (**C**) Flow cytometric analysis of DNA content in hepatoma cells in the presence of hrIL-23. The percentages of cells in each cycle phases were shown. (**D**, **E**) Cells were cultured in serum-free medium containing different concentration of hrIL-23 for 48 h. Then cells were stained with Annexin V and PI to detect the percentage of apoptotic cells by FCM (D) and Bcl-2 level was detected by realtime PCR (**E**). Data were expressed as fold change in Bcl-2 expression in hrIL-23-treated versus untreated cells (arbitrary value = 1). Each value represents the mean ± SD for triplicate samples. ^*^P < 0.05, ^**^P < 0.01, NS, non-significant difference vs negative control (Student *t* test).

To address whether IL-23 could affect the progression of hepatoma cells *in vitro*, the proliferation of cells pre-treated with human recombinant IL-23 (hrIL-23) was assessed by CCK-8 assay and colony-forming assay. As showed in Figure 2A, hrIL-23 promoted the proliferation of HepG2 cells. This promotion peaked at 20 ng/ml. Clonogenic assay also showed that when HepG2 cells were exposed to hrIL-23 for 5–7 days, more and bigger colonies formed (Figure 2B). Furthermore, hrIL-23 promoted progression of the cell cycle at the G1/S transition (Figure 2C). The same results were observed in Huh-7 cell line (Figure 2A-2C). In addition, hrIL-23 increased the expression of Ki-67 at dose-dependent manner (data not shown). Our results confirmed that hrIL-23 promoted proliferation and clone formation of hepatoma cells. The optimal concentration was 20 ng/ml.

As to the effect of hrIL-23 on apoptosis of hepatoma cells, we observed that apoptotic HepG2 cells declined ∼20% from baseline by 5 ng/ml hrIL-23 treatment and continued to decline to 8.4% ± 0.9% by 20 ng/ml hrIL-23, with slight recovery detected by 40 ng/ml hrIL-23 (Figure 2D). Furthermore, mRNA level of anti-apoptosis related gene Bcl-2 was observed to elevate in hrIL-23 treated hepatoma cells (Figure 2E). No obvious changes were observed in the expression of p53 and Survivin in these cells (data not shown).

### hrIL-23 induces motility and invasivity of hepatoma cells

**Figure 3:**
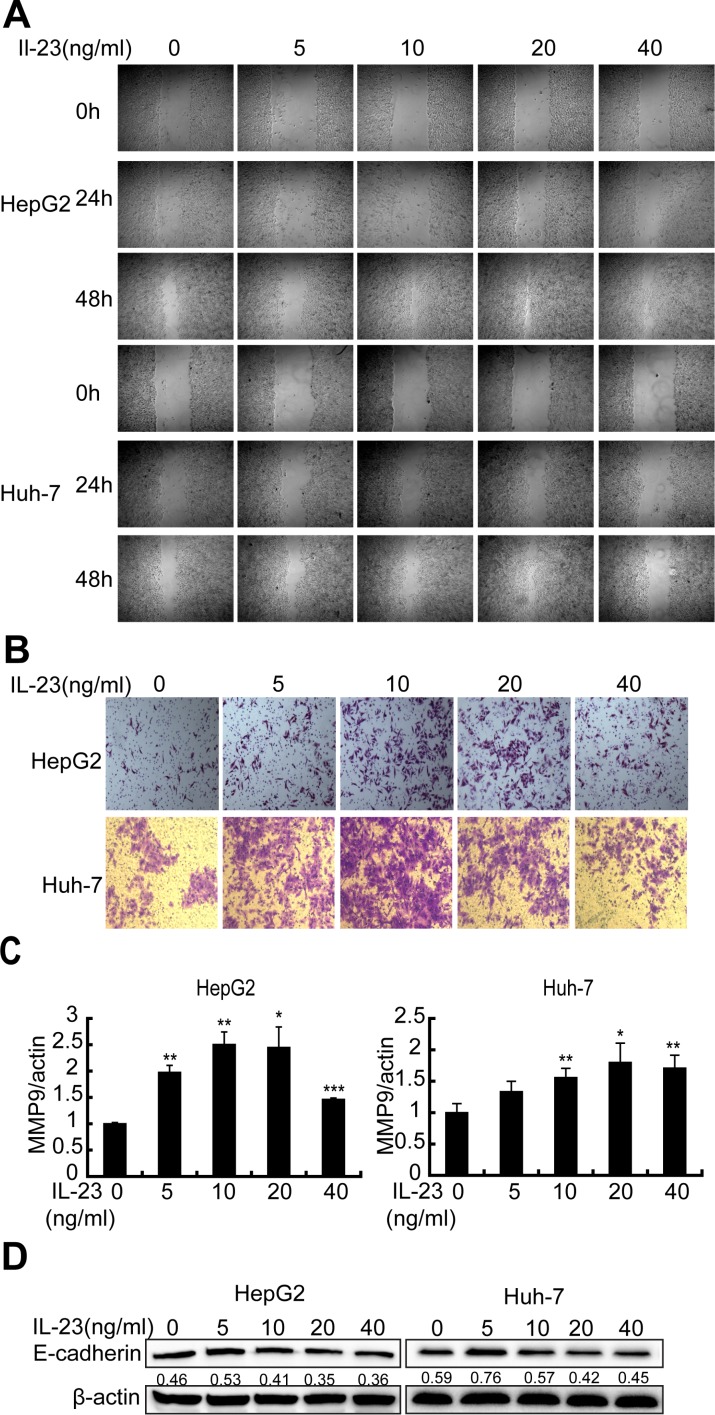
hrIL-23 induces motility and invasivity of hepatoma cells (**A**) Wound healing assays. Cells were plated in 12-well plates and a scratch was made 24 h later. And then cells were cultured in 2% FBS medium containing hrIL-23 for 24 h and 48 h to check wound closure with an inverted microscope. (**B**) Cell motility assays. 1 × 10^5^ HepG2 or Huh-7 cells pretreated with increasing doses of hrIL-23 (0–40 ng/ml) for 24 h were seeded in the upper chamber in serum free medium and 2.5% FBS medium was added to the lower chamber. 24 h later, migrated cells were fixed with 4% paraformaldehyde and then stained with 0.1% Crystal Violet. (**C**) Realtime PCR analysis for MMP9 expression in hrIL-23 treated hepatoma cells. Data were expressed as fold changes in MMP9 expression in hrIL-23-treated versus untreated cells (arbitrary value = 1). Each value represents the mean ± SD for triplicate samples. ^*^P < 0.05, ^**^P < 0.01, ^***^P < 0.001 vs negative control (Student *t* test). (**D**) Western blot showed that the expression of E-cadherin protein in hrIL-23 treated hepatoma cells. The band densitometry readings were normalized to β-actin loading control.

In order to study the biological effects of IL-23 on cellular properties associated with the malignant phenotypes, scratch wound assays were carried out. It was ascertained that 20 ng/ml hrIL-23 pretreated hepatoma cells acquired good motility, but 40 ng/ml hrIL-23 treated cells did not (Figure 3A). The enhancement of hrIL-23 on cell motility and invasivity could be observed by a transwell assay in the presence of matrigel, which mimicks the extracellular matrix microenvironment (Figure 3B).

Transcriptional and translational changes observed coherently in the two lines demonstrated that hrIL-23-induced tumor promoting effects were associated with an EMT gene expression profile: upregulation of invasivity marker matrix metalloproteinase 9 (MMP9) mRNA (Figure 3C) and downregulation of liver-specific and epithelial marker E-cadherin protein (Figure 3D) in HepG2 and Huh-7 cells.

### hrIL-23 increases the proportion of stem/progenitor cells in hepatoma cells

**Figure 4:**
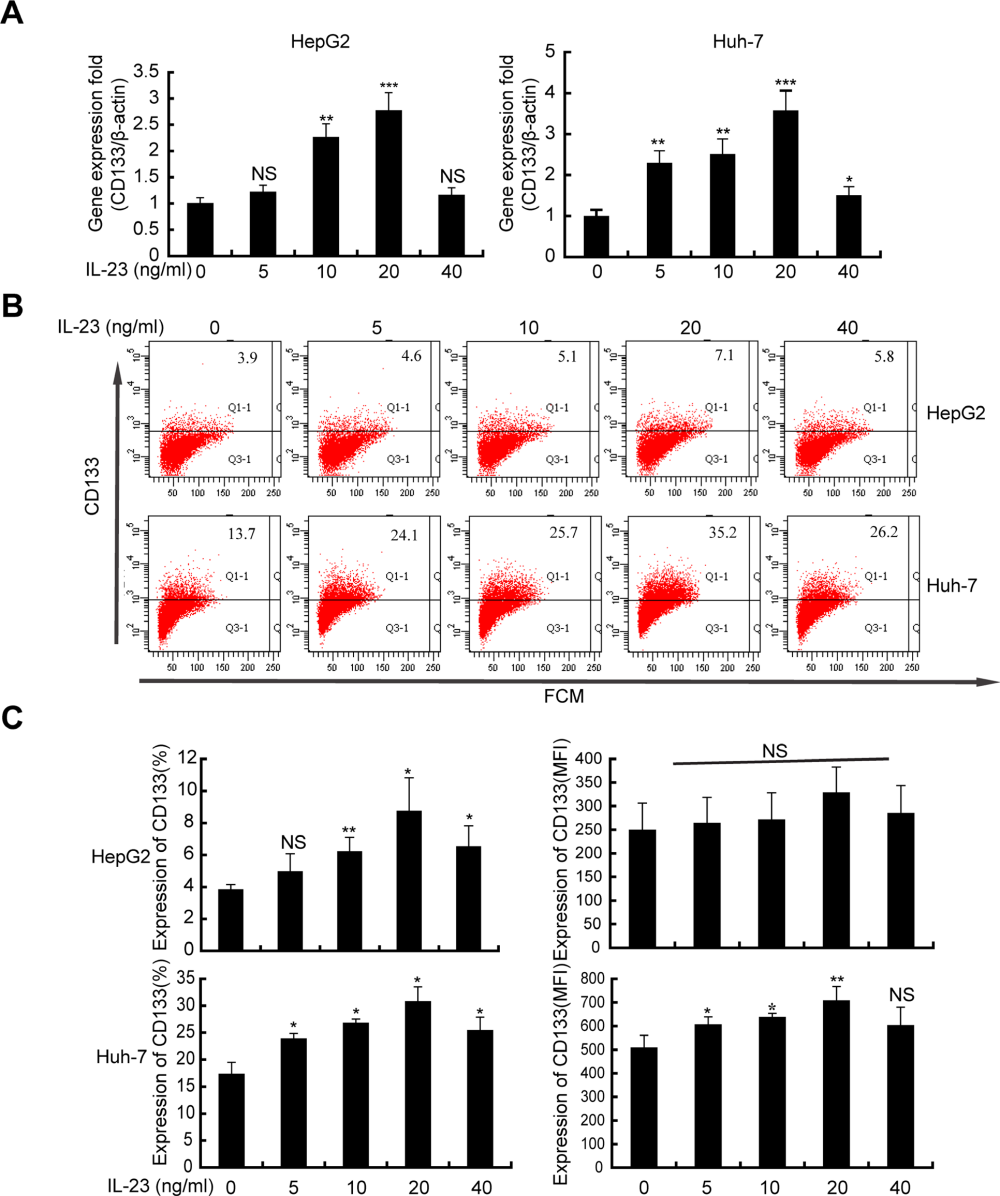
hrIL-23 promotes the “stemness” gene CD133 expression in hepatoma cells Cells were treated with hrIL-23 at different concentrations as indicated for 24 h or 48 h. CD133 expression was analyzed by realtime PCR (**A**) and flow cytometer (**B**). Bar graphs (**C**) are the percentage and MFI of CD133 expression. Each column and bar represents the mean ± SD of three independent experiments. ^*^P < 0.05, ^**^P < 0.01, ^***^P < 0.001, NS, non-significant difference vs negative control.

CD133 [22–24] is a well-known marker for the identification of cancer stem/progenitor cells in HCC. CD133 expression confers malignant potential which may contribute to the survival of HCC cells [25]. To study the effect of IL-23 on CD133 expression, CD133 level was detected by real-time PCR and flow cytometry on hrIL-23 pretreated HepG2 and Huh-7 cells. As showed in Figure 4A, significant upregulation in CD133 mRNA level was detected in both cells. Similarly, flow cytometry confirmed the increment in the mean fluorescence index (MFI) and the proportions of CD133^+^ cells in both cells. These enhancements achieved maximum when cells were pretreated by hrIL-23 at 20 ng/ml (Figure 4B and 4C).

### Attenuation of HNF4α by HBV-related IL-23

**Figure 5:**
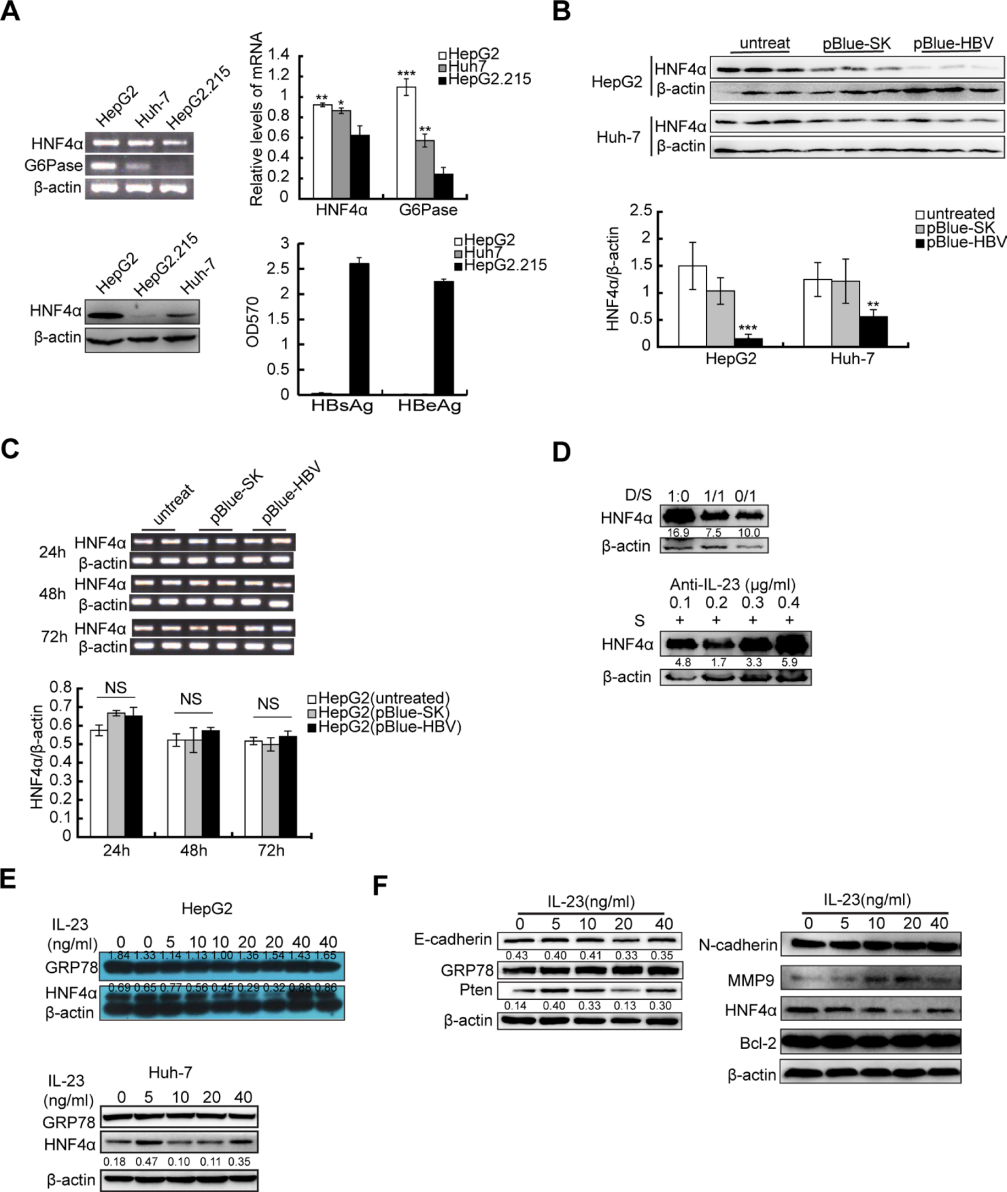
Downregulation of HNF4α by HBV-related IL-23 (**A**) RT-PCR and Western blot show the expression of HNF4α and/or G6Pase in hepatoma cells. ^*^P < 0.05, ^**^P < 0.01, ^***^P < 0.001, NS vs HepG2.215. ELISA shows the levels of HBsAg and HBeAg in culture supernatant of hepatoma cells. (**B**) 72 h after transfection with pBlue-HBV, protein level of HNF4α in hepatoma cells was detected with western blot and counted with bar graph. ^**^P < 0.01, ^***^P < 0.001, vs mock plasmids. (**C**) RT-PCR and photograph showed the mRNA expression of HNF4α in HepG2 transfected with pBlue-HBV for 24 h, 48 h or 72 h. (**D**) Western blot was used to detect the expression of HNF4α in HepG2 cells cultured in DMEM supplemented with different proportions of HepG2.215 supernatant (D/S) or cultured in HepG2.215 supernatant supplemented with different concentration of anti-hIL-23p19 antibody for 24 h. (**E**) Expressions of HNF4α in hrIL-23 treated HepG2 and Huh-7 cells were detected by western blot. (**F**) Expressions of E-cadherin, PTEN, N-cadherin, MMP9, Bcl-2 and HNF4α in hrIL-23 treated HepG2 cells. The WB band densitometry readings were normalized to β-actin loading control. Each column and bar represents mean ± SD.

Hepatocyte nuclear factor 4 alpha (HNF4α) is known as liver-enriched transcription factor [21, 26]. Downregulation of HNF4α is closely related to progress of HCC [27]. Our data showed that the expressions of HNF4α and its target gene G6Pase were severely suppressed in HepG2.215 cells which expressed high concentrations of Hbs and Hbe proteins (Figure 5A). After transiently transfected with plasmids encoding for the full length of HBV genome, HepG2 and Huh-7 cells reduced their HNF4α expression in protein levels (Figure 5B) albeit no changes in their mRNA levels (Figure 5C). When cultured in medium mixed with different proportions of HepG2.215 culture supernatant, HepG2 cells also decreased their HNF4α levels and this decrease was abolished in the presence of anti-IL-23p19 antibody (Figure 5D). These data suggested that HBV infected cells could down-regulate their HNF4α expression, which may be mediated by the secretion of IL-23. To confirm the impact of IL-23 on its expression, HNF4α was detected in hrIL-23-treated hepatoma cells. Western blot showed that HNF4α level gradually decreased as hrIL-23 concentration increased in a certain range (0–20 ng/ml, Figure 5E), as well as the levels of E-cadherin, PTEN but not GRP78. Restoration of HNF4α expression (at 40 ng/ml hrIL-23) could block the reduction of E-cadherin and PTEN (Figure 5F). Contrary to decrease in HNF4α protein level, MMP9 level increased as hrIL-23 concentration increased in a certain range. However, the levels of N-cadherin and Bcl-2 were not altered (Figure 5F).

## DISCUSSION

HBV-related chronic hepatitis is one of major risk factors for HCC [3–5]. Tumor development is accompanied with production of a great number of cytokines, chemokines and growth factors, favoring increased cellular proliferation. Among these, IL-23 plays an important role in chronic inflammation and is the hub of tumor-associated inflammation and tumor immune escape [17, 28]. mDCs and macrophages are the main sources of IL-23. Xia *et al.* [8] reported that the hepatocytes were one of the main cell source that secreted IL-23 in CHB patients. In this paper, our data confirmed that IL-23 was produced by hepatoma cells and its expression was upregulated in HBV-infected cells. This increment was in accordance with previous reports that IL-23 can be induced by HBx and upregulated in the serum and liver tissues of CHB patients [7, 8].

There is a debate about roles of IL-23 in carcinogenesis. Some studies have shown that IL-23 inhibits tumor growth [14–16], and some suggest that IL-23 promotes carcinogenesis [17–20]. Our findings suggested that hrIL-23 enhanced the proliferation of HepG2 and Huh-7 cells in a dose-dependent manner. Furthermore, IL-23 could affect multiple aspects of their malignant phenotypes, such as promoting colony formation, inhibiting apoptosis through enforced expression of BCl-2, inducing motility and invasivity through upregulation of MMP9 and downregulation of E-cadherin, increasing the proportion of stem/progenitor cells. Based on these findings, IL-23 was suggested to participate in the progression of HCC. That could partly explain why chronic infection with HBV is one of the major risk factors for HCC.

As demonstrated in this study, IL-23 promoted carcinogenesis of hepatoma cells. However, this promotion peaked at 20 ng/ml and slightly decreased at 40 ng/ml. Li *et al.* [20] reported that low concentrations of IL-23 bound to the higher affinity IL-23R resulting in promotion of human lung cancer cells proliferation, whereas a high concentration of IL-23 bound to both IL-23R and IL-12Rβ1 resulting in inhibition of their proliferation. Whether the different affinities of the two receptors affect the action of IL-23 on hepatoma cells is worth of our further investigation.

HNF4α, as a master transcriptional regulator of hepatocyte differentiation, is a target gene of cytokines. It is essential for liver development and hepatocyte function [21], and has links to cancer [27, 29]. Substantial evidences had revealed that the expression of HNF-4α was decreased in human HCC compared to the adjacent noncancerous tissues [27, 30, 31]. Loss of HNF-4α facilitates HCC progression [27], whereas introduction of HNF-4α obviously prevented the development of HCC [30, 32]. Engagement of IL-23R by IL-23 results in phosphorylation of tyrosine residues to induce downstream effector molecules for STAT, MAPK, PI3K and NF-kB signaling [11, 33–35]. STAT3 is a key molecule of IL-23 signaling pathway [36, 37] and its activation inhibits expression of HNF4α [38]. Our results manifested that HepG2 and Huh-7 cells showed significantly higher expression of HNF4α than HepG2.215 cells, and introduction of HBV dramatically down-regulated their HNF4α expression. These findings were consistent with previous reports that overexpression of HBx in HepG2 cells inhibited HNF-4α expression, and HNF-4α levels were inversely related with viral proteins both in HBV-infected HepG2.215 cells and HBV positive HCC liver tissues [39, 40]. However, other studies showed that HNF4α plays important roles in transcription and replication of HBV by binding and upregulating the HBV promoter activity [41, 42]. Our results confirmed that HepG2.215 did express lower level of HNF-4α and high amount of HBsAg and HBeAg. It is suggested that the malignant properties of HepG2.215 maybe correlated with loss of HNF4α. Hence, it is worthy of our further investigation to explain how HBV, IL-23 and HNF4α talk with each other to affect HCC progression.

In summary, our results suggested that HBV infection could attenuate the expression of HNF4α via secretion of IL-23. Hence, it is extrapolated that 1) HBV drives infected hepatocytes to produce more inflammatory cytokines, especially IL-23, then 2) IL-23 engages in IL-23R to turn down the expression of HNF4α, and finally 3) inhibition of HNF4α induces progression of hepatocellular carcinoma by increasing ratio of stem/progenitor cells, promoting the proliferation and colony formation of hepatoma cells *in vitro*, and preventing apoptosis and cell cycle arrest of hepatoma cells. Although conflicting reports have assigned HNF4α tumor-promoting role in liver cancer [43], our data support the tumor-suppressing role of HNF4α in line with other reports [30, 32, 38, 44]. Whether IL-23 affects the expression of HNF4α through STAT3 pathway to regulate differentiation of cells needs further study.

Our research still could not answer how HNF4α participates in tumor progression. Hatziapostolou M *et al.* suggested that a HNF4α-miRNA inflammatory feedback loop circuit was essential [38]. F Garibaldi *et al.* [45] inferred that HNF4α directly repressed Snail gene to stabilize the hepatocytic phenotype. Walesky *et al.* suggested that some proliferation-related genes came into play [44]. Downregulation of HNF4α plays a critical role in promoting EMT and affecting apoptosis and cell cycle progression [46]. In this article, we identified that anti-apoptosis related gene Bcl-2, and tumor suppressor protein PTEN participated in this IL-23 and HNF4α mediated tumor progression. The expression change of HNF4α might be positively correlated with E-cadherin, and negatively with MMP9 in IL-23 mediated tumor progression. However, significant work remains to identify the underlying mechanisms involved in tumor progression of HBV related IL-23.

In conclusion, we demonstrated that HBV drove infected hepatoma cells to produce more IL-23. At low concentration, hrIL-23 enhanced malignant properties of hepatoma cell lines HepG2 and Huh-7, which might promote their progression into invasive carcinoma. And this effect could be mediated through attenuation of HNF4α. The findings identified potential targets of interventional strategies for treating hepatitis B patients through manipulation of the IL-23 and HNF4α.

## MATERIALS AND METHODS

### Cell culture and plasmids

Human hepatoma cell lines HepG2, Huh-7, HepG2.215 were obtained from the China Center for Type Culture Collection (Wuhan, China). They were maintained in DMEM medium (Gibco, BRL Co. Ltd, USA) containing 10% fetal bovine serum (Gibco), 100 units/ml penicillin and 100 mg/ml streptomycin (Sigma, St Louis, MO). Cells were cultured at 37° C in a humidified atmosphere of 5% CO_2_.

For analyzing HNF4α expression affected by HBV-related IL-23, HepG2.215 cells were cultured with serum-free medium for 48 h. Then supernatant was collected and mixed with DMEM in different proportions (D/S). After that, HepG2 cells were cultured with D/S or supernatant (S) with IL-23 neutralization antibody (anti-IL-23p19, R&D, Minnesota, USA) for 24 h.

Plasmid pBlue-HBV encoding the full length of HBV was preserved by our lab.

### RNA extraction, reverse transcription, RT-PCR and Real-time PCR

Total RNA was extracted with TRIzol (Invitrogen, California, USA) and reverse transcribed with MMLV reverse-transcriptase (Cwbiotech, Wuhan, China) according to the manufacturer’s protocol. cDNA was amplified as described previously [47, 48]. Equal amounts of cDNA were submitted to real-time PCR in the presence of the SYBR Green qPCR mix (ToYoBo, Osaka, Japan) or RT-PCR in the presence of BioRad Miniopticon (Cwbiotech). The specific primers used were as follows: HNF4α, G6Pase, IL-33, IL-6, TNF, IL-23p19, IL-1β, IL-17, HMGB1, β-actin1, IL-23R, MMP9, CD133, Bcl-2, β-actin2 (Table 1).

**Table 1:** Sequence of specific primers used for RT-PCR and real-time PCR

Gene	Forward	Reverse	Bp
HNF4α	5′-GCCTACCTCAAAGCCATCAT-3′	5′-GACCCTCCCAGCAGCATCTC-3′	275
G6Pase	5′-GCCACATCCACAGCATCTATAA-3′	5′-CCAGAGTCCACAGGAGGTCTAC-3′	129
IL-33	5′-TTCCCTCTGTATAACTGC-3′	5′-AGTGTTTGAGCCTATCGT-3′	283
IL-6	5′-TGAAAGCAGCAAAGAGGC-3′	5′-TGAAAGCAGCAAAGAGGC-3′	259
TNF	5′-CGAGTCTGGGCAGGTCTA-3′	5′-GAAGTGGTGGTCTTGTTGC-3′	199
IL-23p19	5′-GCTTCAAAATCCTTCGCAG-3′	5′-GATCTGAGTGCCATCCTTGAG-3′	119
IL-1β	5′-ACAGTGGCAATGAGGATG-3′	5′-TGTAGTGGTGGTCGGAGA-3′	243
IL-17	5′-CAACCGATCCACCTCACC-3′	5′-AGCCCACGGACACCAGTA-3′	470
IL-23R	5′-ATCGTGAATGAGGAGTTGCC-3′	5′-TGTGCCTGTATGTGTGACCA-3′	470
β-actin1	5′-CTGGGGCGCCCCAGGCACCA-3′	5′-CTCCTTAATGTCACGCACGATTTC-3′	540
HMGB1	5′-TATGGCAAAAGCGGACAAGG-3′	5′-CTTCGCAACATCACCAATGGA-3′	196
MMP9	5′-CGCTACCACCTCGAACTTTG-3′	5′-GCCATTCACGTCGTCCTTAT-3′	196
CD133	5′-ACATGAAAAGACCTGGGGG-3′	5′-GATCTGGTGTCCCAGCATG-3′	200
Bcl-2	5′-CTGGTGGGAGCTTGCATCAC-3′	5′-ACAGCCTGCAGCTTTGTTTC-3′	150
β-actin2	5′-GCAAAGACCTGTACGCCAAC-3′	5′-GTACTTGCGCTCAGGAGGAG-3′	120

### Western blot

Cells were lysed in RIPA buffer containing 1mM protease inhibitors PMSF (Beyotime, Shanghai China). Equal amounts of protein were separated and then blotted with antibodies as follows: antibodies against HNF4α (C19, 1:500), E-cadherin (1:500) and β-actin (1:500) from Santa Cruz Biotechnology (Santa Cruz, USA), anti-GRP78 (1:1000) from Abcam (Cambridge, UK), antibodies against N-cadherin (1:1000), MMP9 (1:1000) and Bcl-2 (1:1000) from Cell Signaling Technology (Beverly, MA, USA) and HRP-conjugated species-specific secondary antibodies (1:2000 dilution, ProteinTech Group, Wuhan, China). After enhanced chemiluminescence reaction (Tiangen, Beijing, China), relative amounts of protein were quantified using Image Gel-Pro analyzer software (Media Cybernetics, Florida, USA).

### Cell proliferation assay

Cell proliferation was assessed using the Cell Counting Kit-8 (CCK8, Zoman Biotechnology, Beijing, China). Briefly, 5 × 10^3^ cells were cultured in DMEM containing less than 1% FBS for 12 h for synchronization. 24 h after supplement of hrIL-23 (Peprotech, New Jersey, USA), CCK-8 solution was added. 2.5 h later, cell proliferation was determined by microplate reader at a wavelength of 450 nm.

### Colony Formation assay

Huh-7 or HepG2 cells (5 × 10^3^) were cultured in DMEM medium supplemented with hrIL-23 and 2% FBS for 5–7 days. Cells were fed fresh medium every 2–3 days. Colonies were fixed with 4% paraformaldehyde and visualized via crystal violet staining.

### Cell cycle analysis

Synchronized cells were exposed to hrIL-23 for 24 h. After fixation, cells were treated with RNAase (10 μg/ml) and then stained with propidium iodide (PI, 10 μg/ml, BD, New York, USA). Cell cycle was analyzed by flow cytometer (LSR II, BD Biosciences) using software Flowjo.

### Apoptosis assay

Cells (1.5 × 10^5^) were incubated in serum-free culture supplemented with hrIL-23 for 48 h. Then cells were stained with Annexin V and PI and analyzed by flow cytometer. Plots from the gated cells showed the populations corresponding to viable (Annexin V^–^PI^–^), early and late apoptotic (Annexin V^+^PI^–^, Annexin V^+^PI^+^) cells.

### Immunofluorescence assay

For detection of IL-23R, cells were cultured with 1:100 diluted goat anti-IL-23R (Abcam) at 4° C and then with 1:100 diluted rabbit anti-goat PE-conjugated secondary antibody (ProteinTech Group, Wuhan, China) at 4° C in the dark. After that, half cells were analyzed by flow cytometer and half cells were further stained with hochest33342 (5 μg/ml, Beyotime) for 10min followed by observation under the fluorescence microscope (LSM 710 and ConfoCor 3, Zeiss, Germany).

For detection of CD133, saponin permeabilized cells were incubated with PE labeled anti-CD133 (Miltenyi Biotec, Germany) and then were analyzed by FACS Calibur.

### Cell culture wound closure assay

For the wound healing assays, cells were plated in 12-well plates and a scratch was made 24 h later. A low serum (2% FBS) culture medium was added to the plates to inhibit cell proliferation. hrIL-23 was supplemented at different concentrations. Wound closure was checked under an inverted microscope 24 h and 48 h later.

### Transwell cell migration and invasion assays

24-well plates (8 μm pore; Corning Inc, NY, USA) were coated with matrigel (100 μg/cm^2^; BD, NJ, USA). hrIL-23-pretreated cells (1 × 10^5^) were then plated in the upper chamber in serum free medium. 2.5% FBS medium was added to the lower chamber. 24 h later, migrated cells were fixed with 4% paraformaldehyde and then stained with 0.1% Crystal Violet.

### Enzyme-linked immunosorbent assay (ELISA)

HBsAg and HBeAg in the culture medium were measured by ELISA kits (Kehua Bioengineering, Shanghai, China), respectively. In Briefly, HepG2, Huh-7 and HepG2.2.15 cells were seeded in 24-well plates at 70–80% confluence. The culture media were collected, removed precipitate after centrifugation for 20 minutes at 2500 rpm and measured levels of HBsAg and HBeAg according to the manufacturer’s instructions.

### Statistical analysis

Values were expressed as means ± SD. Student’s *t*-test was applied to all analyses except for percentage data which were analyzed by Mann-Whitney *U* test using SPSS 17.0 statistical software (SPSS Inc., USA). *P* < 0.05 was considered significant.

## Abbreviations

HCC: Hepatocellular carcinoma
HBV: Hepatitis B virus
HCV: Hepatitis C virus
HBsAg: HBV surface antigen
HNF4α: Hepatocyte nuclear factor 4 alpha
CCK-8: Cell Counting Kit-8
PI: Propidium iodide
MMP9: Matrix metalloproteinase 9
MFI: Mean fluorescence index
ELISA: Enzyme-Linked Immunosorbent Assay
